# The effect of cannabis-derived terpenes on alveolar macrophage function

**DOI:** 10.3389/ftox.2024.1504508

**Published:** 2025-01-31

**Authors:** Patrick M. Greiss, Jacquelyn D. Rich, Geoffrey A. McKay, Dao Nguyen, Mark G. Lefsrud, David H. Eidelman, Carolyn J. Baglole

**Affiliations:** ^1^ Meakins-Christie Laboratories, Montreal, QC, Canada; ^2^ Translational Research in Respiratory Diseases Program at the Research Institute of the McGill University Health Centre, Montreal, QC, Canada; ^3^ Department of Pathology, McGill University, Montreal, QC, Canada; ^4^ Department of Microbiology and Immunology, McGill University, Montreal, QC, Canada; ^5^ Department of Medicine, McGill University, Montreal, QC, Canada; ^6^ Department of Bioresource Engineering, McGill University, Montreal, QC, Canada; ^7^ Department of Pharmacology and Therapeutics, McGill University, Montreal, QC, Canada

**Keywords:** cannabis, terpenes, inflammation, phagocytosis, macrophages

## Abstract

*Cannabis sativa* (marijuana) is used by millions of people around the world. *C. sativa* produces hundreds of secondary metabolites including cannabinoids, flavones and terpenes. Terpenes are a broad class of organic compounds that give cannabis and other plants its aroma. Previous studies have demonstrated that terpenes may exert anti-inflammatory properties on immune cells. However, it is not known whether terpenes derived from cannabis alone or in combination with the cannabinoid ∆^9^-THC impacts the function of alveolar macrophages, a specialized pulmonary innate immune cell that is important in host defense against pathogens. Therefore, we investigated the immunomodulatory properties of two commercially-available cannabis terpene mixtures on the function of MH-S cells, a murine alveolar macrophage cell line. MH-S cells were exposed to terpene mixtures at sublethal doses and to the bacterial product lipopolysaccharide (LPS). We measured inflammatory cytokine levels using qRT-PCR and multiplex ELISA, as well as phagocytosis of opsonized IgG-coated beads or mCherry-expressing *Escherichia coli* via flow cytometry. Neither terpene mixture affected inflammatory cytokine production by MH-S cells in response to LPS. Terpenes increased MH-S cell uptake of opsonized beads but had no effect on phagocytosis of *E. coli*. Addition of ∆^9^-THC to terpenes did not potentiate cytotoxicity nor phagocytosis. These results suggest that terpenes from cannabis have minimal impact on the function of alveolar macrophages.

## Introduction

*Cannabis sativa* L. (Cannabaceae), commonly referred to as marijuana, is a flowering plant that has been used by humans for at least 2,500 years because of its psychoactive and medicinal properties. Today, cannabis is the most-used illicit substance worldwide (Drašar and Moravcova, 2004). While the psychoactive abilities of cannabis are due to the presence of the cannabinoid Δ^9^-tetrahydrocannabinol (Δ^9^-THC), *C. sativa* produces hundreds of additional secondary metabolites including other cannabinoids, flavones, and terpenes. Terpenes are aromatic organic hydrocarbons produced by a variety of plants and some insects. Biologically, terpenes protect plants by repelling insects and herbivores and are responsible for the odor emitted by plants and fruits (Preteroti M. et al., 2023). Terpenes are the primary constituents of essential oils, are used as food additives, and are present in cosmetic products such as soaps and perfumes (Zhou and Pichersky, 2020). Cannabis contains over 200 terpenes, mostly monoterpenes and sesquiterpenes, with differing amounts and variations of the isoprene unit, a 5-carbon hydrocarbon containing at least one double bond, arranged in a head-to-tail formation (Kuzuyama and Seto, 2003). Isoprene is a universal building block and is thus implicated in many metabolic pathways in most living organisms; it forms the side-chains of vitamins A, E and K, leads to the formation of cholesterol, and is a component of the electron transport chain molecule ubiquinone (Kandi et al., 2015). As such, there is incredible diversity in terpene variations, making them the largest group of natural products, with more than 25,000 individual molecules reported (Gershenzon and Dudareva, 2007). Monoterpenes, which contain two isoprene units, are the most common, making up 80% of plant essential oils, and contain well known terpenes such a limonene and pinene, responsible for the flavors and scents of lemons and pine trees, respectively.

The biological response of terpenes in cannabis remains poorly characterized. Most people consume cannabis via inhalation of smoke from a joint or an aerosol generated from heating the dry plant. Thus, terpenes are inhaled upon smoking or vaping along with combustion products. Among the first cells that encounter inhaled chemicals are alveolar macrophages, an innate immune cell responsible for protecting the lungs against foreign pathogens (Ince et al., 2018). Alveolar macrophages are specialized tissue-resident macrophages derived embryonically from the yolk sac. They are responsible for patrolling the luminal side of the lung, recycling surfactant, and protecting against infectious organisms by engulfing pathogens and releasing cytokines (Mosser et al., 2021). The phenotypes of macrophages are said to exist on a spectrum that ranges from inflammatory (classically activated; M1) to anti-inflammatory (alternatively activated; M2) (Joshi et al., 2018). M1 macrophages, typically activated by compounds such as lipopolysaccharide (LPS), release inflammatory cytokines including tumor necrosis factor (TNF) and interleukins such as IL-6 and IL-1β. In contrast, the M2 phenotype can be induced by cytokines IL-4 and IL-13, and these macrophages release anti-inflammatory counterparts IL-10 and transforming growth factor beta (TGF- β) (Zhang and An, 2007).

Several studies have demonstrated immunomodulatory properties of terpenes on macrophage functions, including phagocytosis, migration and release of pro-inflammatory cytokines (Chi et al., 2013; Younis, 2020; Schulze-Osthoff et al., 1997). However*,* no research has been performed on the effects of cannabis-derived terpenes on alveolar macrophage function. Therefore, we investigated whether two mixtures of cannabis terpenes affected the functional responses of MH-S cells, a murine alveolar macrophage cell line*.* Furthermore, terpenes are claimed to potentiate the biological activity of ∆^9^-THC, a phenomenon named the entourage effect. Although a popular term in the cannabis industry, very little evidence exists to demonstrate an entourage effect between terpenes and cannabinoids. Thus, we also sought to understand whether ∆^9^-THC influenced the pharmacological properties of terpenes. We quantified MH-S cell apoptosis, inflammatory mediator production and phagocytosis in response to cannabis terpene mixtures with and without ∆^9^-THC. Our data show that terpenes have minimal effects on MH-S cell function and did not act synergistically with ∆^9^-THC. Thus, claims of the beneficial properties of terpenes need to be interpreted with caution until additional studies emerge to support the notion that cannabis-derived terpenes affect biological responses in the lungs and other organs.

## Materials and methods

### Cell culture

MH-S cells were obtained from American Type Culture Collection (ATCC; Manassas, VA, United States) and cultured in RPMI (Roswell Park Memorial Institute) 1640 media (WISENT Inc, Saint-Jean Baptiste, Canada) containing 10% fetal bovine serum (FBS) (WISENT Inc), gentamicin (WISENT Inc), antibiotic-antimycotic (A/A; WISENT Inc) and 2-mercaptoethanol (Millipore Sigma, Burlington, MA, United States). Cells were treated with the following: Terpene Mix A (CRM40755; Sigma-Aldrich, St. Louis, United States), Terpene Mix B (CRM40937; Sigma-Aldrich) (Table 1), LPS (LPS 0111: B4; Sigma-Aldrich), the toll-like receptor 2 (TLR2) agonist Pam3CSK4 (tlrl-pms; InvivoGen, San Diego, United States), ∆^9^-THC (ISO60157; Cayman Chemical, Ann Arbor, United States) or the appropriate controls which included methanol (MeOH) or serum-free (SF) RPMI 1640. All cells were incubated in humidified chambers at 37°C and exposed to 21% O_2_ and 5% CO_2_.

**TABLE 1:** A list of terpenes in terpene mix A and B; ± represents racemic mixture (equal proportions) of chemical enantiomers.

Terpene Mix A	Terpene Mix B
(R)-(+)-limonene	p-Cymene
(−)-α-Cedrene	trans-3,7-dimethyl-2,6-octadien-1-ol
(±)-Camphene	Dipentene
(+)-Pulegone	(−)-Pin-2 (10)-ene
p-mentha-1,4-diene	(1S)-3,7,7-Trimethylbicyclo [4.1.0]hept-3-ene
Geranyl acetate	Linalool
3,7,7-trimethylbicyclo [4.1.0]hept-3-ene	
3,7,11-Trimethyldodeca-1,6,10-trien-3-ol	
pin-2 (3)-ene	

### MTT assay

MH-S cells were plated at 4.7 × 10^4^ cells/cm^2^ in a sterile, flat-bottomed 96 well plate (Thermo Fisher Scientific) in RPMI 1640 containing 10% FBS. Forty-eight h later, cells were treated with SF-RPMI 1640, varying concentrations of Terpene Mix A and Terpene Mix B in SF-RPMI 1640, MeOH, PBS (positive control), and 3 μg/mL of ∆^9^-THC (Sigma Aldrich) with and without Terpene Mix A and B for 24 h. Then, a 5 mg/mL solution of 3-(4,5-dimethylthiazol-2-yl)-2,5 diphenyl tetrazolium bromide (MTT; Sigma-Aldrich M-2128) in PBS was prepared and 10 μL of this solution was added to each well. Following a 4-h incubation period, the plates were centrifuged at 800 RPM for 5 min, the supernatant was discarded, and the contents of each well were resuspended in 200 µL of dimethyl sulfoxide (DMSO; Sigma Aldrich). Ten minutes later, plates were read on an iMark microplate reader (Bio-Rad Laboratories, Canada) using Microplate Manager Software Version 6 at 570 nm.

### Quantitative reverse transcription PCR (qRT-PCR)

Cells were plated at 4 × 10^4^ cells/cm^2^ in a 6-well plate (Thermo Fisher Scientific) and upon reaching 80% confluency, 24–48 h later, were pre-treated with 1 μg/mL of Terpene Mix A or Terpene Mix B. One hour later, 0.1 μg/mL LPS was added for 24 h. RNA was isolated using Aurum Total RNA Mini Kit (Bio-Rad Laboratories, Canada) in accordance with instructions from the manufacturer. RNA was quantified using Nanodrop 1000 Spectrophotometer Infinite M200 Pro (TECAN, Mannedorf, Switzerland). Reverse transcription was accomplished with iScript Reverse Transcription Supermix (Bio-Rad Laboratories, Canada) to a final concentration of 10 ng/mL. Primer sequences for the genes were: *18S* (forward) CGG​AAA​ATA​GCC​TTC​GCC​ATC​AC, (reverse) ATC​ACT​CGC​TCC​ACC​TCA​TCC​T; *Tnf* (forward) CTA​TGT​CTC​AGC​CTC​TTC​TC, (reverse) GGG​AAC​TTC​TCA​TCC​CTT​T; *Il1b* (forward) GGA​CAT​GAG​CAC​CTT​CTT, (reverse) CCT​GTA​GTG​CAG​TTG​TCT​AA and *Il6* (forward) CCAGAGTCCT TCAGAGAGATACA, (reverse) CCT​TCT​GTG​ACT​CCA​GCT*TAT​C.* qPCR was done by combining 1 μL of cDNA and 0.25 μL of forward and reverse primers with SsoFast Evagreen (Bio-Rad Laboratories). Amplification was performed using a CFX96 Real-Time PCR Detection System (Bio-Rad Laboratories). Thermal cycling was initiated at 95°C for 3 min followed by 39 cycles of denaturation at 95°C for 10 s and annealing at 55°C or 52°C for *S18*/*il6* and *il1b* and *tnf*, respectively, for 5 s. RNA expression was calculated using the ΔΔCt method and results were expressed as the log_2_ fold change normalized to housekeeping gene *18S.*

### Cytokine quantification

Following treatments for 24 h, the cell supernatant was collected and a Mouse Cytokine Array Proinflammatory Focused 10-plex (MDF10) Assay was conducted (Eve Technologies, Alberta, Canada). Only the results are shown for IL-1β, IL-6 and TNF.

### Measurement of phagocytosis of opsonized latex beads

MH-S cells were plated in a 6-well plate (Thermo Fisher Scientific) and treated with SF-RPMI 1640, 100 ng/mL Pam3CSK4 (positive control), MeOH (vehicle) or 1 μg/mL of terpene mixes A or B; 24 h later, IgG-coated beads were added to each well at a final volume of 10 μL per well (dilution 1:100 in SF-RPMI 1640) and incubated for 4 h. Medium was then aspirated, cells were washed 2x with PBS and detached using 1 mL of Accutase Cell Detachment Solution (Innovative Cell Technologies, United States). Alveolar macrophages were then stained with Amcyan fixable viability dye (Thermo Fisher Scientific) for 30 min. Cells were acquired using a FACSCanto flow cytometer (BD Biosciences, Canada) and analyzed using FlowJo software version 10.

### Preparation of fluorescent mCherry *Escherichia coli*

XL1 Gold *E. coli* containing the replicative plasmid pUC18-miniTn7T2.1::PA1/04/03-mCherry, which constitutively expresses mCherry fused to a synthetic Lac promoter (Zhao et al., 2013), was grown on LB agar plates (Thermo Fisher Scientific) containing 10 mg/mL gentamicin. The mCherry plasmid was isolated from these colonies using a plasmid miniprep kit (GeneJet Plasmid Miniprep Kit K0503, Thermo Scientific) according to the manufacturer’s instructions. The plasmid concentration was measured using a NanoDrop Microvolume Spectrophotometer (Thermo Fisher Scientific) and subsequently transformed into K12 *E. coli* via electroporation as previously described (Dower et al., 1988). Electroporated K12 *E. coli* was then plated on LB agar containing 10 mg/mL gentamicin for positive selection. Successful transformation of the mCherry plasmid was confirmed by measuring the fluorescence at an excitation wavelength of 587 nm and an emission wavelength at 620 nm as well as the optical density at 600 nm of 200 μL samples of cultures from five individual colonies grown in Miller LB broth (Thermo Fisher Scientific) at 37°C, shaken at a duration of 5 s and an amplitude of 1 mm once an hour for 18 h using the Infinite 200 pro plate reader (TECAN, Zürich, Switzerland). The colony presenting the highest fluorescence to optical density ratio was plated on LB agar containing 10 mg/mL gentamicin for 24 h before storage in LB with 15% v/v glycerol at −80°C.

### Measurement of phagocytosis of *Escherichia coli*

MH-S cells were plated in a 6-well plate and treated with SF-RPMI 1640, MeOH (vehicle) and 1 μg/mL of terpene mixes A or B with or without 3 μg/mL of ∆^9^-THC. Twenty-four hours later, mCherry-expressing K12 *E. coli* was applied to the wells at an MOI of 50. To synchronize phagocytosis, plates were centrifuged at 700 RPM for 3 min before incubation at 37°C and 5% CO_2_ for 75 min. After incubation, cells were washed three times with ice cold PBS to stop degradation in the phagolysosome and to wash away residual extracellular bacteria. Cells were then detached with 1 mL Accutase Cell Detachment Solution, stained with Amcyan fixable viability dye for 30 min, and fixed with 4% paraformaldehyde in PBS (Thermo Fisher Scientific) for 15 min. Cells were acquired using the LSRFortessa X-20 flow cytometer (BD Biosciences; Canada) and analyzed using FlowJo software version 10 (Supplemental Figure S1).

### Annexin V and propidium iodine (PI) staining

Cells were plated and treated as previously described. Twenty-four h after treatment, cells were detached with 1 mL Accutase Cell Detachment Solution and simultaneously stained with 5 μL APC-Annexin V and 3 μL PI for 15 min. Cells were then acquired using the FACS Canto flow cytometer (BD Biosciences, Canada) and data were analyzed using FlowJo software version 10 (Supplemental Figure S2).

### Statistical analysis

Unless otherwise stated, statistical analysis was performed using a one-way analysis of variance (ANOVA) with Bonferroni’s multiple comparison test to assess differences between the untreated control group and any treatment groups. Statistical analyses were performed on GraphPad Prism 10 (version 10.0.3; GraphPad Software Inc, San Diego, CA, United States). Results are presented as mean ± standard error of the mean (SEM). Statistical significance was considered where the *P* value <0.05.

## Results

### Alveolar macrophage inflammatory genes and proteins are not affected by cannabis-derived terpenes

**FIGURE 1:**
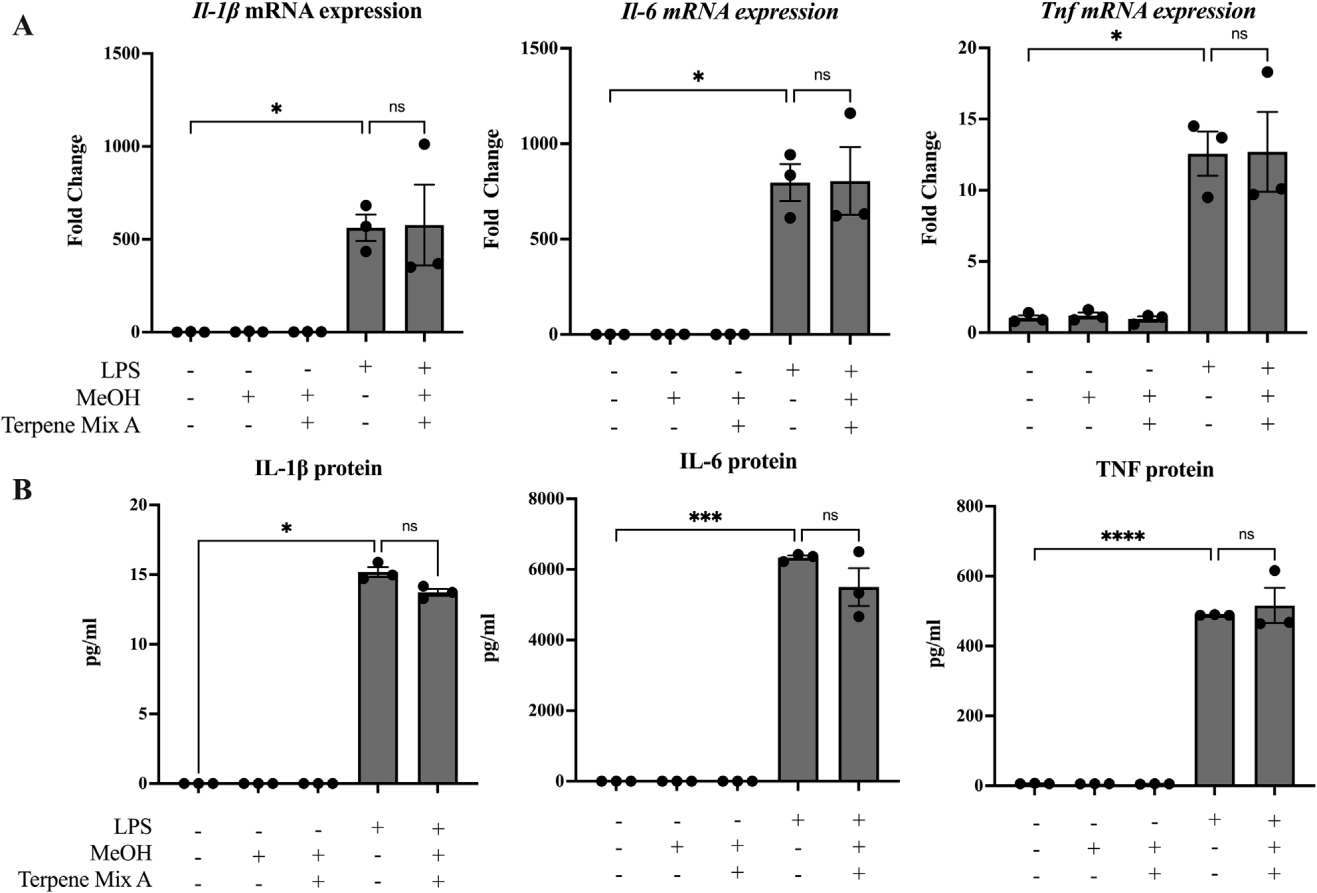
Terpene Mix A does not attenuate inflammatory cytokine production in response to LPS. MH-S cells were pretreated for 1 h with Terpene Mix A followed by the addition of LPS for 24 h before measurement of **(A)** mRNA and **(B)** protein for IL-1β, IL-6 and TNF. Results are expressed as the mean ± SEM of three independent experiments. Kruskall Wallis multiple comparisons test was applied in panel B due to a protein concentration of 0 for untreated condition; ns = not significant, *P < 0.05, ***P < 0.001, ****P < 0.0001.

**FIGURE 2:**
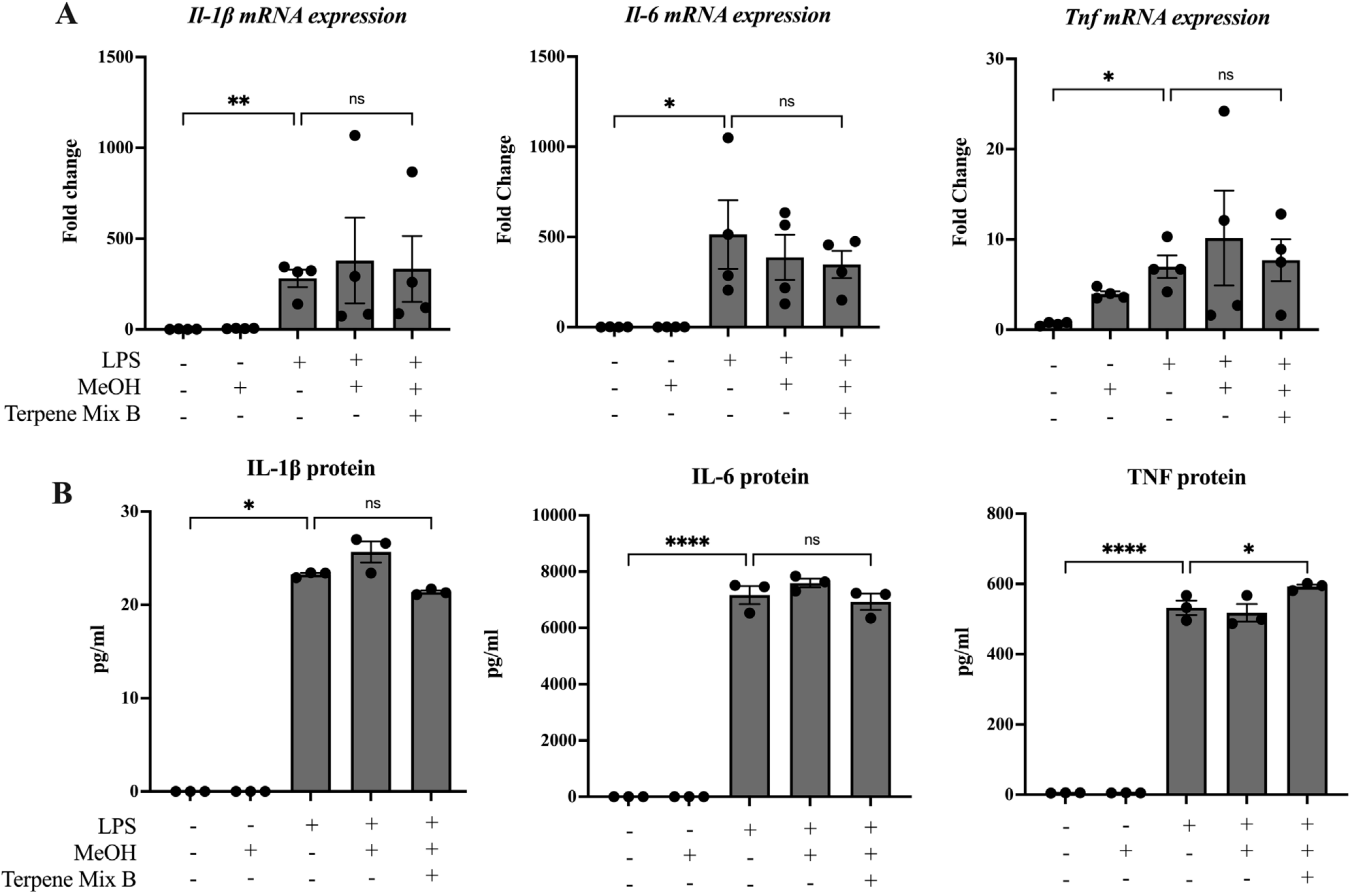
Terpene Mix B does not attenuate inflammatory cytokine production in response to LPS. MH-S cells were pretreated for 1 h with Terpene Mix B followed by the addition of LPS for 24 h before analysis of **(A)** mRNA and **(B)** protein for IL-1β, IL-6 and TNF. Results are expressed as the mean ± SEM of 3–4 independent experiments. Kruskall Wallis multiple comparisons test was applied in panel B due to a protein concentration of 0 for untreated samples; ns, not significant, *P < 0.05, ****P < 0.0001.

Some studies have suggested that terpenes found in cannabis and other plants are anti-inflammatory (Rufino et al., 2015; Gallily et al., 2018). Because most people consume cannabis via inhalation, we hypothesized that cannabis-derived terpenes would dampen the ability of alveolar macrophages to mount an inflammatory response. To be sure that any observed effect was not confounded by alterations in cellular viability, we first determined the maximal concentration of each terpene mixture that did not significantly reduce cell survival to be 1 μg/mL (Supplemental Figure S3); this concentration was used in all subsequent experiments. We then analyzed these cannabis-derived mixtures on inflammatory cytokine mRNA and protein expression of MH-S cells in response to LPS as previously described (Preteroti et al., 2023b). IL-1β, IL-6 and TNF were selected as our primary outcomes because of their potent transcriptional induction by LPS as well as their downregulation by ∆^9^-THC (Rossol et al., 2011). As expected, LPS significantly increased the mRNA and protein expression of IL-1β, IL-6 and TNF after 24 h (Figures 1, 2). Neither Terpene Mix A (Figure 1A) nor Terpene Mix B (Figure 2A) alone affected the transcription of *Il1b, Il6,* or *Tnf.* Neither Terpene Mix A (Figure 1A) or Terpene Mix B (Figure 2A) affected LPS-induced gene expression. Although LPS also significantly increased the release of IL-1β, IL-6, and TNF protein after 24 h, this was not attenuated by either terpene mixture (Figures 1B, 2B). Further, the protein levels of GM-CSF, IFN**γ,** IL-2, IL-4, IL-10, IL-12p70 and MCP-1 were also not affected by terpene treatment (data not shown). Thus, cannabis-derived terpenes have minimal impact on inflammatory mediator production in response to LPS in MH-S cells.

### Terpenes from cannabis cause a ligand-dependent increase in MH-S cell phagocytic capability

**FIGURE 3:**
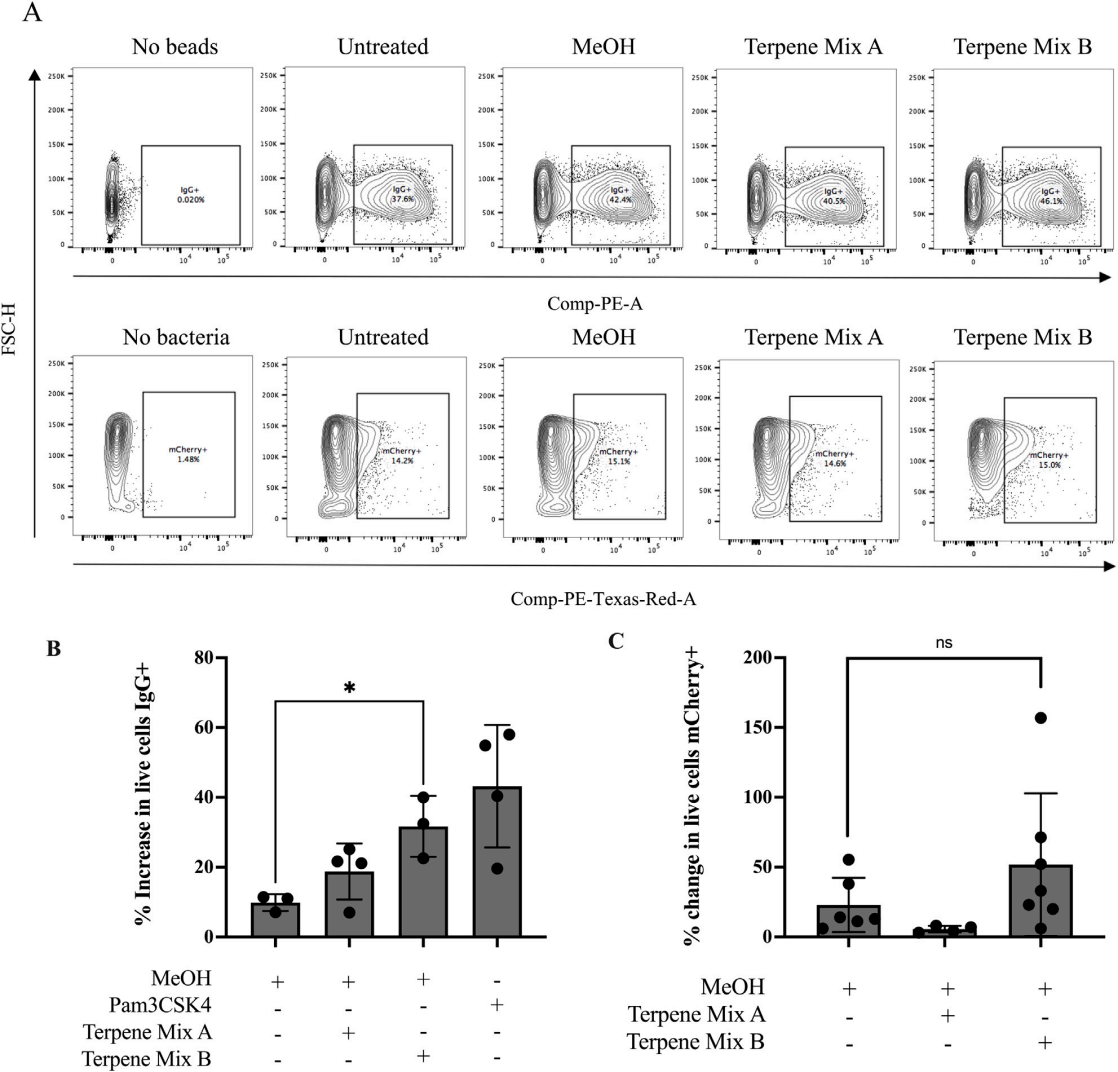
Terpene Mix B increases phagocytosis against opsonized beads. **(A)** Representative gating strategies for quantification of phagocytic cells using flow cytometry. **(B)** Terpene Mix B increased the percentage of phagocytic macrophages by approximately 30% when challenged with fluorescently labelled latex beads opsonized with IgG. **(C)** Neither terpene mixture significantly affected phagocytosis of mCherry-expressing *Escherichia coli*. Results are expressed as the mean ± SEM of 3–6 independent experiments. ns, not significant, *P < 0.05.

A primary function of alveolar macrophages is to phagocytose inhaled pathogens and foreign debris (Joshi et al., 2018). Hence, we analyzed the phagocytic capability of MH-S cells in response to treatment with cannabis-derived terpenes. First, we quantified the percentage of phagocytic cells by using flow cytometry to measure the uptake of latex beads opsonized with IgG and conjugated to a phycoerythrin (PE) fluorophore (Figure 3A). As a positive control, we used Pam3CSK4, a TLR2 agonist which has been previously described as a potent enhancer of phagocytosis against opsonized ligands (Sigola et al., 2016). Pam3CSK4 increased the percentage of MH-S cells that took up the IgG-conjugated beads by approximately 30% (Figure 3B). While Terpene Mix A did not significantly affect uptake of the fluorescent beads (Figure 3), Terpene Mix B significantly increased IgG internalization (Figure 3B). Thus, terpenes can selectively increase the ability of alveolar macrophages to phagocytose opsonized particles.

Macrophages can recognize and phagocytose multiple types of pathogens, and opsonized ligands are one type of particle that binds to the fragment crystallizable receptor gamma (FcGammaR) to stimulate internalization. Hence, we extended our analysis by using a common non-virulent laboratory strain of *E. coli* which is detected by scavenger receptors on macrophages. We transformed wild type K12 *E. coli* with a plasmid encoding for a constitutively expressed fluorescent mCherry protein to track association of the bacteria to MH-S cells (Supplemental Figure S4). Because we did not perform a gentamicin treatment following co-incubation of MH-S cells with bacteria, our assay did not exclude bacteria stuck to the outside of the MH-S cell membrane, and thus complete internalization could not be confirmed. However, pretreatment with either cannabis-derived terpene mixture A or B before incubation with these bacteria had no significant effect on the ability of MH-S cells to phagocytose *E. coli* (Figures 3A, C). Therefore, these results suggest that terpenes do not affect the ability of MH-S cells to phagocytose bacteria.

### Cannabis-derived terpenes do not potentiate the effects of ∆^9^-THC in MH-S cells

**FIGURE 4:**
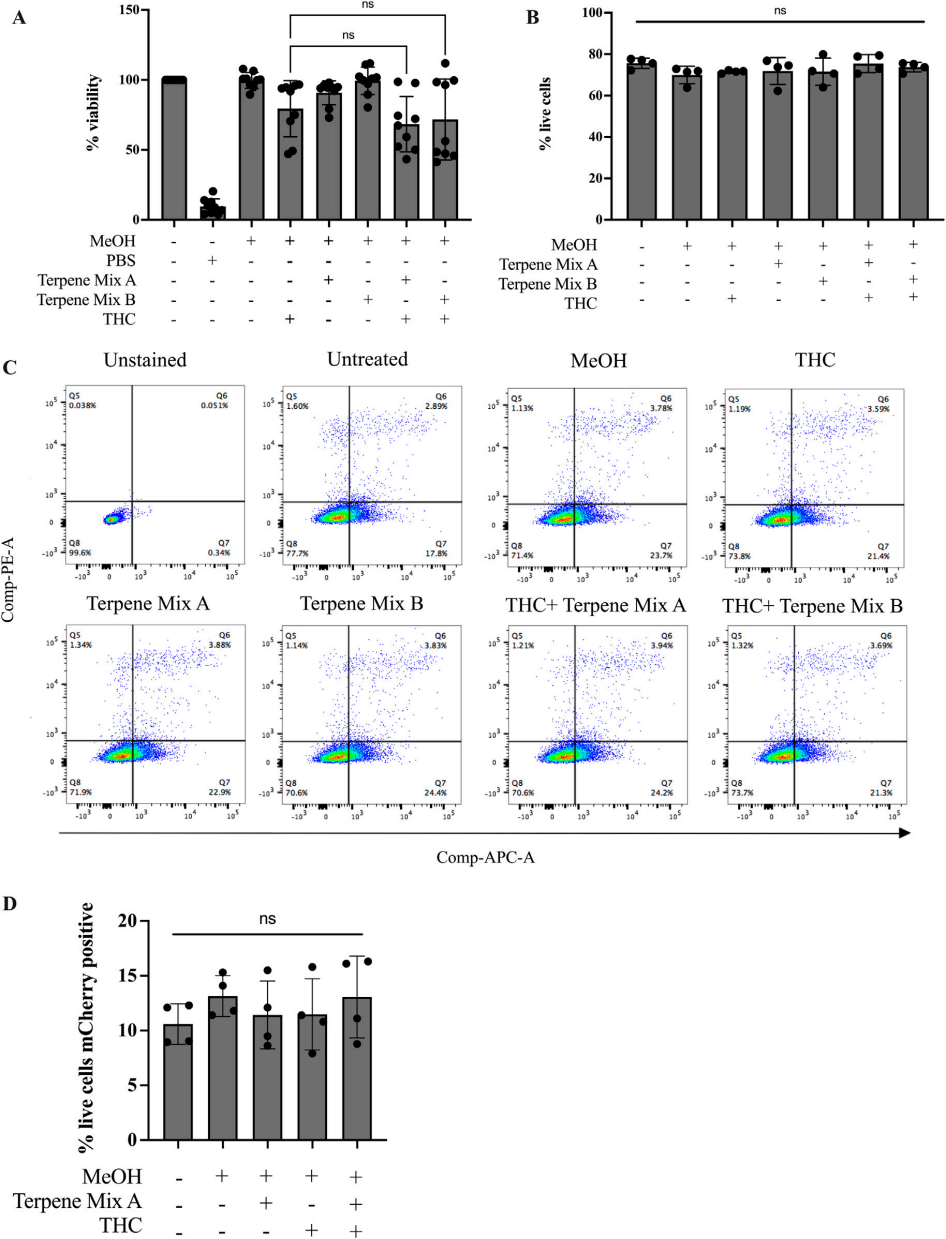
Terpenes do not potentiate the effects of Δ^9^-THC. Treatment of MH-S cells with terpenes in combination with Δ^9^-THC had no additive effect as measured by MTT assay **(A)** or Annexin-V PI staining assay **(B)**. **(C)** Representative gating strategy for Annexin-V PI assay as measured by flow cytometry. **(D)** Terpene Mix A in combination with Δ^9^-THC did not affect phagocytosis of mCherry-expressing *E. coli.* Results are expressed as the mean ± SEM of 4–9 independent experiments; ns, not significant.

Our data above imply that cannabis-derived terpenes have minimal effect on alveolar macrophage function. However, a prevailing concept is that cannabis-derived terpenes may exert a synergistic effect with ∆^9^-THC, which is commonly referred to as the entourage effect (Russo, 2011). However, there exists very little evidence to suggest that terpenes in fact synergize with ∆^9^-THC to potentiate its effects, and no information exists for the entourage effect in alveolar macrophages. Therefore, we utilized ∆^9^-THC, together with the cannabis-derived terpene mixtures, and assessed cell viability and phagocytosis. We used concentrations of 3 μg/mL and 1 μg/mL ∆^9^-THC and Terpene Mix A or B (Supplemental Figure S3) (Preteroti et al., 2023b). When ∆^9^-THC was included with Terpene Mix A or B, there was no significant effect on cell viability when compared to each treatment individually as measured by MTT assay (Figure 4A). As a complement, we also performed the Annexin-V/PI staining, which allows the differentiation between healthy, apoptotic and necrotic cells (Miller, 2004). Here, there was no difference in apoptosis or necrosis between cells treated with ∆^9^-THC and/or cannabis-derived terpenes (Figures 4B, C). Finally, we measured the phagocytic capability of MH-S cells. Treatment with Terpene Mix A nor ∆^9^-THC alone significantly affected MH-S cell phagocytosis (Figure 4D). There was also no difference when Terpene Mix A was combined with ∆^9^-THC (Figure 4D). These results suggest that cannabis-derived terpenes do not enhance the effects of ∆^9^-THC in MH-S cells. Overall, these results show that terpenes have minimal impact on the function of alveolar macrophages, resident pulmonary cells that are important in protecting the lungs against inhaled pathogens.

## Discussion

A growing list of countries, including Canada, Germany and Mexico, have legalized or decriminalized cannabis for both recreational and medical use. Many studies attribute the therapeutic potential of cannabis to the anti-inflammatory effects of cannabinoids such as cannabidiol (CBD) and ∆^9^-THC on immune cell functions (Borrelli et al., 2009; Carmona-Hidalgo et al., 2021; Jeon et al., 1996; Ribeiro et al., 2015; Suryavanshi et al., 2022). There is also interest into the potential biological effects of cannabis-derived terpenes at alleviating inflammation. However, little is known about the immunomodulatory effects of terpenes, particularly on the function of alveolar macrophages, which are among the first cell types to encounter inhaled cannabis. Alveolar macrophages are key innate immune cells specialized in responding to environmental stimuli and protecting against pathogens. In this study, using a murine model of alveolar macrophages, we demonstrate that terpenes derived from cannabis do not significantly modulate the ability of alveolar macrophages to mount an inflammatory response or phagocytose foreign bacteria.

Many pulmonary immune pathologies such as chronic obstructive pulmonary disease (COPD) and cystic fibrosis (CF) are characterized by excessive and prolonged release of inflammatory mediators by lung cells such as alveolar macrophages (Bezerra et al., 2023; Cantin et al., 2015). The release of inflammatory cytokines can further act on innate immune cells such as neutrophils, eosinophils and epithelial cells to produce reactive oxygen species (ROS), which, if not appropriately regulated, results in oxidative stress which can damage the lung parenchyma (Moldoveanu et al., 2008). Further, alveolar macrophages are crucial in limiting the severity of lung damage caused by inhaled pathogens by rapidly engulfing bacteria and virus during the early stages of infection (Pribul et al., 2008; Kooguchi et al., 1998). Thus, any perturbation of alveolar macrophage function could have deleterious effects on the host. For these reasons, we evaluated whether terpenes derived from cannabis could alter the immune response in MH-S cells, thereby providing insight into the potential health implications of inhaling cannabis products. However, we found that terpenes did not suppress the release of inflammatory mediators induced by LPS and marginally affected their propensity to phagocytose opsonized ligands. It is therefore likely that the presence of cannabinoids, particularly THC and CBD, are the phytochemicals in cannabis that control immunological function. THC and CBD can dampen the inflammatory response in numerous cell types, including alveolar macrophages (Preteroti et al., 2023b; Coffey et al., 1996; Srivastava et al., 1998). These findings suggest that any immunomodulatory properties of cannabis on the lungs is likely not due to the presence of terpenes but are likely due to cannabinoids or other compounds.

Our results contrast with published data on the effect of many terpenes, including limonene, pinene, linalool and caryophyllene, on immune function. These terpenes, which were also present in the terpene mixtures we used, have been shown to attenuate the release of pro-inflammatory cytokines by macrophages of the peritoneum *in vivo*, as well as *in vitro* using the mouse macrophage cell line RAW264.7 (Rufino et al., 2015; Yoon et al., 2010; Andrade-Silva et al., 2016; Islam et al., 2020). This discrepancy between our results and these could be due to various reasons, including the concentrations of the terpenes. Many studies have used individual terpenes for treatment with typical concentrations varying between 10 and 500 μg mL^-1^. We chose to utilize lower concentrations of terpene mixtures at a maximal non-toxic dose of 1 μg mL^-1^ of each terpene within the mixture. The differences in concentrations between our study and others could thereby influence biological outcomes such as inflammatory mediator production and phagocytosis. In addition, no previous study has investigated the effects of terpenes on alveolar macrophages, which have a distinct phenotype and origin compared to other tissue-resident macrophages. This could include the expression of proteins important in controlling macrophage biology. Along these lines is previous work from our group which revealed that MH-S cells lack the expression of adenosine receptor A2a, which is present on other macrophage types including RAW264.7 cells (Skopál et al., 2023). While adenosine receptors are typically associated with pain signaling in the CNS (Zhou et al., 2023), their activation in macrophages has also been shown to reduce inflammatory cytokine production in response to LPS (Devi et al., 2023). Although the data is limited, terpenes limonene, pinene, humulene and geraniol have all been described as ligands of the A2a receptor (Schwarz et al., 2022; LaVigne et al., 2021), suggesting that a lack of inflammatory suppression in our study could be due to the absence of A2aR in MH-S cells. However, further work must be conducted to comprehensively elucidate the receptor-mediated effects of terpenes.

Another aspect of macrophage biology that is important to consider is phagocytosis. However, available information regarding the effect of terpenes on the phagocytic capability of macrophages is equivocal, with evidence suggestive of both increases and decreases in phagocytosis, a discrepancy that may depend on the terpene itself, the dose administered as well as the type and origin of macrophage used in the study (de Carvalho et al., 2021; Hamada et al., 2002; Del Toro-Arreola et al., 2005; da Franca Rodrigues et al., 2015; Carvalho et al., 2017; Giovannini et al., 2016). In our study, we report that only Terpene Mix B, (but not Terpene Mix A), increased the phagocytic capability of MH-S cells in a target-dependent manner. Terpene-treated MH-S cells were more phagocytic against beads opsonized with IgG whereas the internalization of *E. coli* was not affected. These data suggest that terpenes such as linalool and p-cymene, which were present only in Terpene Mix B, may be acting on receptors whose signaling pathways intersect with processes of Fc-**γ** receptor-mediated phagocytosis. An important signalling molecule involved in Fc-**γ** receptor-mediated phagocytosis is Rac1, a GTPase that is responsible for proper cytoskeleton rearrangement necessary for engulfment of opsonized particles (Uribe-Querol and Rosales, 2020). Agonism of the cannabinoid 2 receptor (CB_2_R), present on MH-S cells, has been shown to activate Rac1 (Basu and Dittel, 2011). Terpenes share structural similarities to cannabinoids and may be able to activate CB_2_R (LaVigne et al., 2021; Cavalli and Dutra, 2021). Hence, CB_2_R-mediated Rac1 activation by Terpene Mix B could explain the increase in phagocytosis against IgG-opsonized beads and could explain why neither terpene mixture induced a change in phagocytic capability against *E. coli.*

Although the work done on investigating the immunological effects of terpenes is important for public health reasons in the context of cannabis legalization, there are limitations to the model used in the present work. This includes the fact that cannabis is most often inhaled from smoking, a process during which THC is decarboxylated, transforming the molecule into its biologically active form. The result of burning or heating cannabis to various temperatures during its consumption may significantly alter its chemical composition, including that of the terpenes. In fact, terpenes react strongly with oxygen, especially at high temperatures, and the resultant smoke may contain a broad range of isoprenoid products, many of which are known toxicants (Meehan-Atrash et al., 2021). Therefore, future studies should utilize inhaled models of exposure to best reflect the conditions in which terpenes are consumed by cannabis users.

To conclude, our study demonstrates that terpenes which reflect the chemical profile of *C. sativa* have minimal effects on MH-S cells, a murine model of alveolar macrophages. Furthermore, they do not act synergistically with ∆^9^-THC to impact cell viability or alter phagocytic capability in MH-S cells, dispelling common claims of an entourage effect existing between terpenes and cannabinoids. These findings imply that terpenes are not likely to contribute to pulmonary immune suppression caused by inhaling cannabis.

## Data Availability

The raw data supporting the conclusions of this article will be made available by the authors, without undue reservation.
