# Supplementary material for: The effect of cannabis-derived terpenes on alveolar macrophage function
Source: Front Toxicol. 2025 Jan 31;6:1504508. doi: 10.3389/ftox.2024.1504508 (PMC11825813; doi:10.3389/ftox.2024.1504508)
Supplement: Supplementary file 1 [file DataSheet1.docx]

Supplementary Material


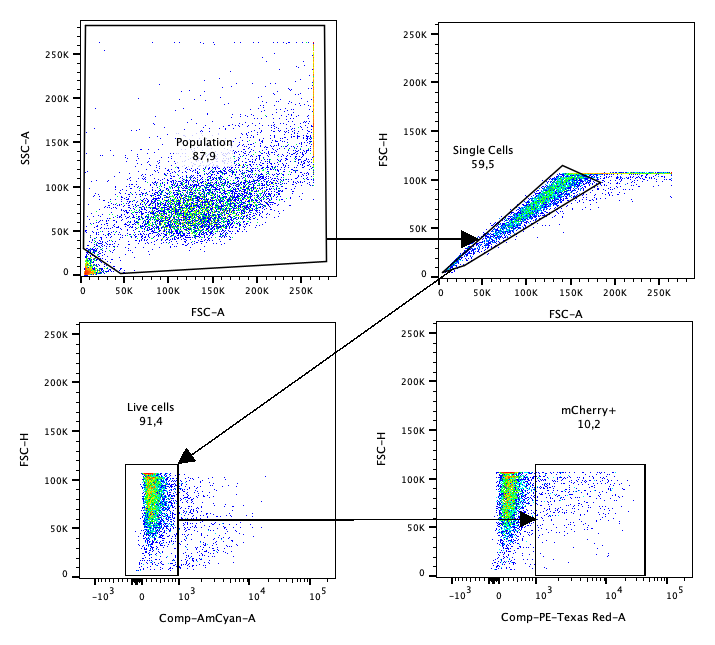


**Supplemental Figure 1.** Gating strategy for phagocytosis assay with using MH-S alveolar macrophages and mCherry+ *E. coli*.

**
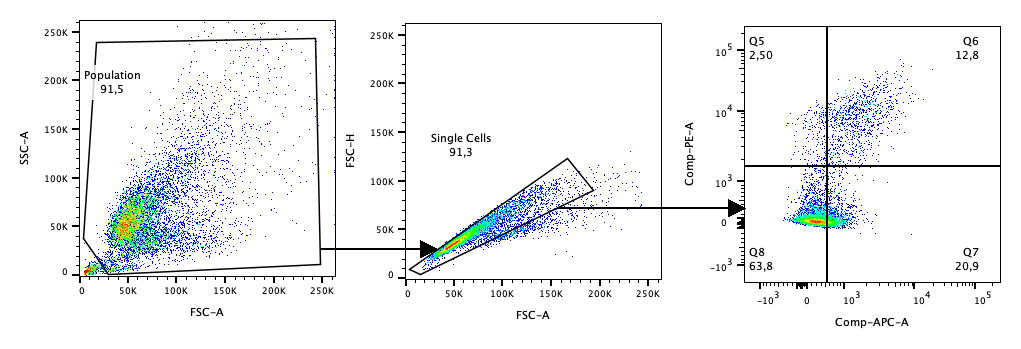
**

**Supplemental Figure 2.** Gating strategy for Annexin-V PI assay. Q6 and Q7 represent late and early apoptotic cells, respectively, while Q5 represents necrotic cells and Q8 contains live cells.

**Supplemental Figure 3.** Terpene mixes A and B have maximal non-toxic doses of 5µg ml^-1^ and 1µg ml^-1^, respectively, as demonstrated by MTT assay. Results are expressed as the mean ± SEM of 3-4 independent experiments; ns, not significant, **P < 0.01, ***P < 0.001.

**Supplemental Figure 4.** Generation of an mCherry-expressing strain of K12 E. coli. **A)** Clones 1 (left) and 4 (right) demonstrated an increasing fluorescence at 620nm as their optical densities increased over time, suggesting successful uptake of the mCherry plasmid. **B)** Fluorescence at 620nm divided by the optical density measured over time, representing relative intensity of mCherry in each sample. Each graph represents one independent experiment.
